# Social and territorial inequalities in the mortality of children and adolescents due to COVID-19 in Brazil

**DOI:** 10.1590/0034-7167-2021-0482

**Published:** 2022-08-08

**Authors:** Rivaldo Mauro de Faria, Leonardo Bigolin Jantsch, Eliane Tatsch Neves, Camila Freitas Hausen, Amanda Peres Zubiaurre de Barros, Graciela Dutra Sehnem, Marina Jorge de Miranda

**Affiliations:** I Universidade Federal de Santa Maria. Santa Maria, Rio Grande do Sul, Brazil; II Universidade Federal de Santa Maria. Palmeira das Missões, Rio Grande do Sul, Brazil; III Ministério da Saúde, Secretaria de Vigilância em Saúde. Brasília, Distrito Federal, Brazil

**Keywords:** Coronavirus Infections, Severe Acute Respiratory Syndrome, Epidemiology, Mortality, Pediatric Nursing., Infecciones por Coronavirus, Síndrome Respiratorio Agudo Grave, Epidemiología, Mortalidad, Enfermería Pediátrica., Infecções por Coronavírus, Síndrome Respiratória Aguda Grave, Epidemiologia, Mortalidade, Enfermagem Pediátrica.

## Abstract

**Objective:**

To analyze the mortality rate of COVID-19 among children and adolescents aged 0 to 14 years.

**Methods:**

Ecological and exploratory study of children’s mortality rate by COVID-19 in Brazil, from February to October 2020. The study used the Severe Acute Respiratory Syndrome database to collect the data and made the analysis using descriptive spatial statistics by age and race/color classification.

**Result:**

The mortality rate due to COVID-19 represented 1.34 deaths per one hundred thousand in the total group evaluated. The age group with the highest frequency and mortality rate was 1 to 4 years of age. There is a higher frequency of deaths in the brown and Indigenous population.

**Conclusion:**

The distribution of deaths due to COVID-19 is unequal in the national territory, and there is a wide variation in the mortality rate by age and race/color groups.

## RESUMO

**Objetivo:**

Analisar a taxa de mortalidade por COVID-19 entre crianças e adolescentes de 0 a 14 anos.

**Métodos:**

Estudo ecológico e exploratório da taxa de mortalidade de crianças por COVID 19 no Brasil, no período de fevereiro a outubro de 2020. A coleta foi feita utilizando-se o Banco de Dados de Síndrome Respiratória Aguda Grave, e a análise foi realizada com recurso à estatística espacial descritiva, por classificação etária e de raça/cor.

**Resultados:**

A taxa de mortalidade por COVID-19 representou 1,34 mortes a cada 100 mil no grupo total avaliado. A faixa etária com maior frequência e taxa de mortalidade foi de 1 a 4 anos. Há maior frequência de óbitos na população parda e indígena.

**Conclusão:**

A distribuição de óbitos por COVID-19 é desigual no território nacional, e há grande variação da taxa de mortalidade por grupos etários e de raça/cor.

## RESUMEN

**Objetivo:**

Analizar la tasa de mortalidad por COVID-19 entre niños y adolescentes de 0 a 14 años.

**Métodos:**

Estudio ecológico y exploratorio de la tasa de mortalidad de niños por COVID-19 en Brasil, en el período de febrero a octubre de 2020. La recolecta fue realizada utilizándose el Banco de Datos de Síndrome Respiratorio Agudo Grave, y el análisis fue realizado con recurso a la estadística espacial descriptiva, por clasificación etaria y de raza/color.

**Resultados:**

La tasa de mortalidad por COVID-19 representó 1,34 muertes a cada 100 mil en el grupo total evaluado. La franja etaria con mayor frecuencia y tasa de mortalidad fue de 1 a 4 años. Hay mayor frecuencia de óbitos en la población parda e indígena.

**Conclusión:**

La distribución de óbitos por COVID-19 es desigual en el territorio nacional, y hay grande variación de la tasa de mortalidad por grupos etarios y de raza/color.

## INTRODUCTION

COVID-19, a clinical disease caused by Severe Acute Respiratory Syndrome Coronavirus 2 (SARS-CoV-2), is already the cause of the greatest health crisis of this century and the most severe epidemiological event in the globalized world^(1)^. Brazil registered its first case on February 22, 2020 and confirmed 5,323,630 cases and 155,900 deaths until October 22 of that year^(2)^. As in other countries, the mortality rate is higher among the elderly and in the population with comorbidities, especially hypertension, obesity, and diabetes^(2)^. Studies have shown profound social inequalities in COVID-19 mortality when classified by racial groups and economic conditions^(3-4)^. In Brazil, there are also significant territorial inequalities in mortality from COVID-19, with rates twice as high in the North Region compared to the South Region, for example^(2)^.

Epidemiological studies on outcomes in patients with COVID-19 from the United States of America, Europe, and China state that 80% of deaths occurred in adults over 65 years of age, and the elderly, over 85 years, had more severe outcomes. Epidemiological data, until then, report little and, in some places, are nonexistent about mortality in children and adolescents^(5)^. European data highlight a low prevalence of deaths in people under 60 years of age and almost absent in children and adolescents^(6)^. The study recognized that children rarely experience severe forms of the disease; however, even with a low prevalence of symptomatic/infected (0.18%), they are also responsible for the spread of the disease^(7-8)^.

Pediatric patients with COVID-19 have a simple transmission mode, either through close contact with infected adults or exposure to epidemic areas. Although fever, dry cough, and mild pneumonia are common manifestations, almost half of patients have no evident symptoms or abnormal radiological findings. The proportion of asymptomatic cases indicates the difficulty of identifying pediatric patients without clear epidemiological information. This finding suggests a dangerous situation if community-acquired infections occur^(9)^.

In the pediatric population, COVID-19 mortality and severity rates are low: they usually evolve as mild or even asymptomatic flu-like symptoms^(6,10-11)^. There is also no evidence of congenital infection, although more accurate assessments are still needed to determine whether the intrauterine transmission is present^(12-13)^. Studies of demographic variations have shown that hospitalization is higher among children under one year of age^(14-15)^, although there is still a lack of evaluations of sociodemographic and regional variables in cases of deaths associated with COVID-19. Due to the brief time and characteristics of the disease, its implications on the development of the child are still inaccurate, as well as the indirect consequences related to control actions (unemployment, income reduction, access to prenatal services, among others).

In Brazil, there is still not a broad epidemiological study that evaluates the impact of COVID-19 on the infant, children, and adolescents’ mortality. The country knows truly little about the absolute number of these deaths. Underreporting and low coverage of exams hamper the understanding of the dimension of the problem, given the existing underreporting framework already evident for other childhood problems^(16)^. Therefore, this study aims to contribute to a first dimensioning of territorial inequalities in the mortality of children and adolescents due to complications derived from COVID-19 in Brazil, as well as to evaluate the variations by age groups and race/color.

## OBJECTIVE

To analyze the mortality rate of COVID-19 among children and adolescents aged 0 to 14 years of age, considering geographical variations by age and race/color.

## METHODS

### Ethical aspects

Considering the use of secondary data evaluated at the country scale, it was not necessary to submit this study to the evaluation by the Research Ethics Committee, according to the regulatory standards for research with human beings in Brazil.

### Design, period, and place of study

It is a mixed-type ecological, descriptive, and exploratory study, guided by the STROBE tool, conducted with all deaths associated with children and adolescents aged 0 to 14 years in Brazil due to complications derived from COVID-19. The study performed the analysis for the 27 Units of the Federation using data collected from the Severe Acute Respiratory Syndrome (SARs) database ^(17)^.

### Population

It is a population study since it included all deaths of children and adolescents aged 0 to 14 years in Brazil, registered as Severe Acute Respiratory Syndrome, and etiologically classified as COVID-19.

### Study protocol

The data were collected by the main study’s researcher using a simple own instrument created for this purpose, which contained characterization variables such as skin color, age group, and state of residence, later inserted into the database created in an Excel spreadsheet. The Influenza Epidemiological Surveillance System (SIVEP-Gripe), created in 2009 for the control of H1N1, collected this information. In 2020, this system also incorporated the registration of COVID-19 cases. The data collected refer to February 16 to October 19, 2020, when the country had already registered 843,666 cases of SARS. Of these, 217,506 evolved to death (25.8%), and 148,454 (17.6%) of these deaths had an etiological classification of COVID-19. The analysis period coincides with the registration of the first case of COVID-19 in Brazil and corresponds to the most recent date for data collection for this study. The study justifies the use of this database because it contains the variables “age” and “race,” which do not appear in the general reports released on the COVID-19 control platform of the Ministry of Health [https://infoms.saude.gov.br/extensions/covid-19_html/covid-19_html.html].

For this purpose, the study also collected data from the preliminary results of the Mortality Information System (SIM) for the year 2020, as it has already incorporated the International Classification of Diseases (ICD-10) for COVID-19 (code B34.2). However, the result of this aggregated database represented a smaller universe of deaths (n = 136,480) than those presented in the SARS control system for the same period. It may occur due to the nature of the systems, which are updated and released periodically according to their specific protocols. Therefore, because it presents a broader scope (n = 148,545), the investigation was maintained using the SIVEP-Gripe data. This data is available at https://opendatasus.saude.gov.br/dataset?tags=SRAG, and the collection and classification, according to selected variables, are made using the data dictionary, available at the same address.

### Criteria of inclusion and exclusion

The study included all deaths from COVID-19 in the population from 0 to 14 years of age and excluded all deaths from other causes, regardless of age.

### Analysis of results and statistics

The research performed the analyses by age classification, race/color, and Unit of the Federation of residence. The age assessment was divided into four groups, considering as a classification criterion the specificities of child health^(17)^ and the classification made by the Brazilian Institute of Geography and Statistics (IBGE): children under one year old; children between 1 and 4 years old, childhood and preschool; children between 5 and 9 years old, preschool and school; and children in the transition to adolescence, and adolescents, the period between 10 and 14 years. This classification also allowed to dimension the deaths concerning the resident population projected by IBGE for the year 2020^(18)^.

The evaluation by race/color was conducted considering the SARS monitoring system’s specific classification in white, black, yellow, brown, and Indigenous people. For the correct typification of cases, SIVEP-Gripe provides a data dictionary, which was used to disaggregate the variables, not only by race/color but also by age and place of residence.

The mortality rate of COVID-19 was given by the relationship between the number of cases and the population residing in the same place (Unit of the Federation) and calculated in the same period. IBGE projections are performed only by age groups with five-year intervals and not by individualized ages. Therefore, the study disassociated the age group from 0 to 4 years of age projected by IBGE in 2020 (less than one year and from 1 to 4 years old) and used (*wi* ) the population growth rate of 0 to 4 years old between 2010 (*P*1 ) and 2020 (*P*2 ) as weight or correction value. In Equation 1, the weight (*wi* ) was applied for each disaggregated age group in the year 2010 (*Xn*1) and added to the population of the same period to achieve the correction with the projected growth rate in 2020.


$$P_{C}=\frac{w_{i}X_{i}}{100}+X_{i}$$

Being *wi* given by the equation:


$$w_{i}=\frac{P_{n_{2}}-P_{n_{1}}}{P_{n_{1}}}*100$$

In which:

*Pc*: corrected age group or race population

*wi*: growth rate used for correction

*Xn*1: population of the disaggregated age or race to be corrected in the period *n*1

*Pn*1: age group population to be corrected in the period *n*1

*Pn*2: age group population to be corrected in the period *n*2

Since the IBGE also does not project by race/color, the study used this same equation for the correction of the population to calculate the mortality rate for each group evaluated. However, in this case, the population (*P*1 and *P*2) is represented by the total scope of the evaluated group, which is from 0 to 14 years of age.

The empirical Bayesian method was applied to investigate possible random rate fluctuations, which is something usual in population-based studies involving areas (Units of the Federation, for example)^(19)^. As these are large population scopes (country scale), the results did not indicate significant variations that would recommend adjusting the rate. Therefore, the study chose to use the crude rate to judge it as adequate and to avoid the creation of an estimated rate for Units of the Federation where no death occurred in the analyzed period (the Bayesian corrects it but also determines an expected rate based on the country’s average, creating, in this case, an abstract value that we try to avoid in this work).

The study calculated the relative distribution as per the relationship between deaths due to COVID-19 in each Unit of the Federation (27) and the total sample evaluated of deaths due to COVID-19 in the same age group evaluated (0 to 14 years). The calculation of the 99% confidence interval for population proportion was applied to ensure the accuracy of the indicator^(20)^.

After calculating the rates, the data were entered into a Geographic Information System using the ArcGIS 10.8 (Esri) program and the IBGE cartographic base on the 1:200,000 scale. This setting also created the tables and maps used in the results presented in this study. The data analysis programs were ArcGIS 10.8 itself and Excel, from the Office 2016 package.

## RESULTS

The relative distribution and mortality rate by age group are shown in Table 1.

**Table 1:** Absolute frequency, frequency relative, and mortality rate from COVID-19 in children and adolescents aged 0 to 14 years in Brazil, Brazil, 2020

	Population^*^	n	%	TM^†^
<1	2,899.940	56	9.5	1.93
1-4	11,830.360	250	42.4	2.11
5-9	14,650.284	159	26.9	1.09
10-14	14,805.480	125	21.2	0.84
Total	44,186.064	590	100.0	1.34

* *Population projected by IBGE, 2020. ^†^Mortality rate: 1/100mil*.

In the analysis by age group, the group with the highest number and relative proportion of deaths was that of 1 to 4 years (42.4%). This group also with the highest mortality rate (2.11/100 thousand). Children under five years of age represent about 52% of the total deaths due to COVID 19 in the population studied, as well as evidence of the highest mortality rates.

Regarding the distribution of deaths by race/color (Table 2), the highest frequency is in the brown population.

**Table 2:** Absolute and relative frequency of deaths of COVID-19 by age and color/race classes in Brazil, Brazil, 2020

Color/Race	Absolute frequency (n)					Absolute frequency (%)				
	< 1	1-4	5-9	10-14	Total	< 1	1-4	5-9	10-14	Total
White	9	42	28	32	111	16.1	16.8	17.6	25.6	18.8
Black	1	6	4	4	15	1.8	2.4	2.5	3.2	2.5
Yellow	1	0	2	1	4	1.8	0.0	1.3	0.8	0.7
Brown	23	135	85	66	309	41.1	54.0	53.5	52.8	52.4
Indigenous people	3	16	8	4	31	5.4	6.4	5.0	3.2	5.3
Unknown	19	51	32	18	120	33.9	20.4	20.1	14.4	20.3
Total	56	250	159	125	590	100.0	100.0	100.0	100.0	100.0

* *Population projected by IBGE, 2020.*

The brown population also has the second-highest mortality rate, emphasizing the most frequent age group (1-4 years) (Table 3). In the analysis of the ethnic group with the highest mortality rate, there is the Indigenous population, with emphasis on the age group from 1 to 4 years of age, with 18.42 deaths per one hundred thousand inhabitants. Regarding the relative and absolute frequency in the number of deaths, the Indigenous population is the race with the third-highest number of deaths, followed by the brown and white populations, respectively.

**Table 3:** COVID-19 mortality rate by age and color/race classes in Brazil, Brazil, 2020

Color/Race	Mortality rate per 100 thousand inhabitants				
	< 1	1-4	5-9	10-14	Total
White	0.58	0.75	0.44	0.52	0.56
Black	0.91	1.02	0.46	0.4	0.58
Yellow	4.57	0	1.46	0.72	0.99
Brown	1.91	2.48	1.19	0.89	1.46
Indigenous people	14.17	18.42	8.19	4.89	10.78

* *Population projected by IBGE, 2020.*

**Figure 1:**
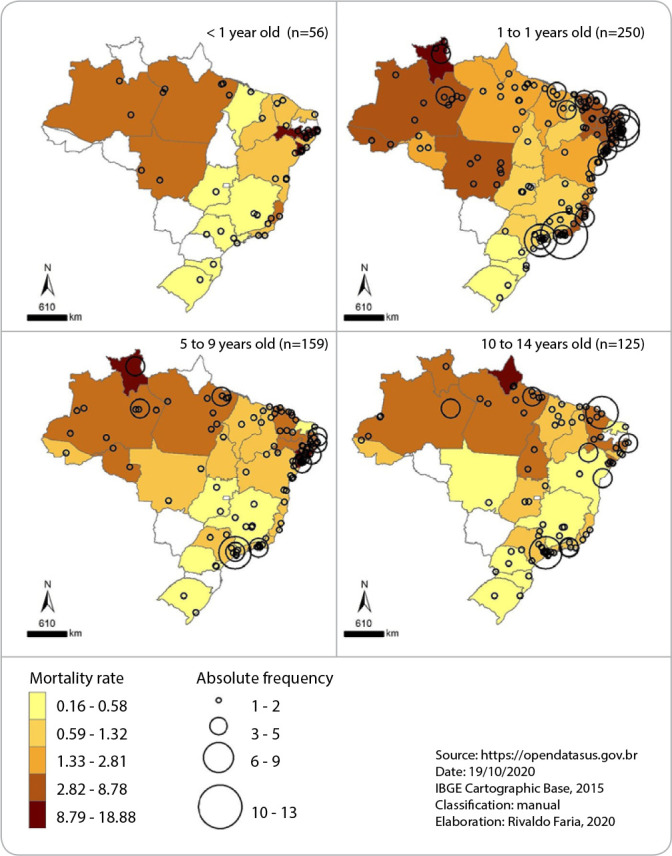
Brazilian distribution of mortality rate and absolute frequency of deaths from COVID 19, Brazil, 2020

The geographic distribution is presented in Table 4 and Figure 1 by frequency and mortality rate per state.

**Table 4:** Absolute frequency and mortality rate from COVID-19 among children and adolescents per Unit of the Federation (UF) in Brazil, Brazil, 2020

UF	Age group							
	Absolute and relative frequency of deaths				Mortality rate per 100 thousand inhabitants			
	< 1	1-4	5-9	10-14	< 1	1-4	5-9	10-14
Acre	0 (0)	2 (0.8)	1 (0.6)	1 (0.8)	0.0	3.0	1.2	1.1
Alagoas	1 (1.8)	7 (2.8)	9 (5.7)	2 (1.6)	2.0	3.5	3.4	0.7
Amazonas	3 (5.4)	12 (4.8)	11 (7)	6 (4.8)	3.8	3.7	2.7	1.5
Amapá	0 (0)	1 (0.4)	0 (0)	3 (2.4)	0.0	1.6	0.0	3.7
Bahia	3 (5.4)	14 (5.6)	12 (7.6)	4 (3.2)	1.5	1.7	1.2	0.4
Ceará	3 (5.4)	20 (8.1)	10 (6.4)	12 (9.7)	2.3	3.8	1.6	1.8
Distrito Federal	0 (0)	0 (0)	0 (0)	0 (0)	0.0	0.0	0.0	0.0
Espírito Santo	2 (3.6)	7 (2.8)	2 (1.3)	2 (1.6)	3.5	3.0	0.7	0.7
Goiás	1 (1.8)	5 (2)	2 (1.3)	3 (2.4)	1.0	1.2	0.4	0.6
Maranhão	1 (1.8)	13 (5.2)	4 (2.5)	5 (4)	0.9	2.7	0.7	0.8
Minas Gerais	2 (3.6)	7 (2.8)	5 (3.2)	4 (3.2)	0.8	0.7	0.4	0.3
Mato Grosso do Sul	0 (0)	0 (0)	0 (0)	0 (0)	0	0	0	0
Mato Grosso	2 (3.6)	9 (3.6)	2 (1.3)	1 (0.8)	3.6	4.0	0.7	0.4
Pará	5 (8.9)	15 (6)	13 (8.3)	15 (12.1)	3.6	2.6	1.8	1.9
Paraíba	0 (0)	13 (5.2)	6 (3.8)	0 (0)	0.0	5.7	2.2	0.0
Pernambuco	18 (32.1)	35 (14.1)	18 (11.5)	10 (8.1)	13.6	6.3	2.5	1.4
Piauí	1 (1.8)	2 (0.8)	2 (1.3)	3 (2.4)	2.2	1.0	0.8	1.2
Paraná	0 (0)	1 (0.4)	2 (1.3)	3 (2.4)	0.0	0.2	0.3	0.4
Rio de Janeiro	3 (5.4)	27 (10.9)	14 (8.9)	12 (9.7)	1.4	3.0	1.3	1.1
Rio Grande do Norte	1 (1.8)	8 (3.2)	1 (0.6)	1 (0.8)	2.1	4.2	0.4	0.4
Rondônia	0 (0)	3 (1.2)	3 (1.9)	0 (0)	0.0	2.7	2.2	0.0
Roraima	0 (0)	9 (3.6)	3 (1.9)	1 (0.8)	0.0	18.9	5.4	1.9
Rio Grande do Sul	1 (1.8)	2 (0.8)	2 (1.3)	2 (1.6)	0.7	0.4	0.3	0.3
Santa Catarina	1 (1.8)	2 (0.8)	0 (0)	2 (1.6)	1.0	0.5	0.0	0.4
Sergipe	4 (7.1)	12 (4.8)	14 (8.9)	5 (4)	12.3	8.8	8.2	2.8
São Paulo	4 (7.1)	21 (8.5)	20 (12.7)	25 (20.2)	0.7	0.9	0.7	0.8
Tocantins	0 (0)	1 (0.4)	1 (0.6)	2 (1.6)	0.0	1.0	0.8	1.5

* *População projetada pelo IBGE, 2020.*

The study showed that the geographical distribution of the number of childhood deaths due to COVID-19 is unequal in the national territory. There is a higher frequency of deaths in the Southeast, North, and Northeast region, especially in the age group older than one year. States such as São Paulo and Rio de Janeiro had the highest number of deaths, but the TM trend did not follow the absolute numbers. The mortality rate is higher in the northern states (especially Roraima and Pará) and Northeast (especially the States of Pernambuco and Sergipe) in all age groups analyzed. The States of Pernambuco, Roraima, and Sergipe had the highest mortality rates due to COVID-19 in the pediatric population.

The State of Mato Grosso do Sul and the Distrito Federal were the only States of the Federation that did not register deaths from COVID-19 in any of the age groups studied. The lowest mortality rates were recorded in the states of the South Region (Paraná, Santa Catarina, and the Rio Grande do Sul), followed by Minas Gerais and São Paulo in the Southeast Region.

## DISCUSSION

In the last 30 years, Brazil has recorded considerable advances in the decrease in infant mortality (under one year) and childhood (under five years), as mortality rates reduced from 85 to 14 deaths per thousand children born alive. This improvement reflects the better health conditions available in a country, and such achievements are mainly due to the Basic Health System (SUS) implemented in the 1980s, with the strengthening of public policies. It is also associated with economic growth, the reduction of income disparities, the increase in women’s schooling, the decrease in birth rates, and the transfer of income^(21)^. Despite this significant reduction, there was an increase in childhood mortality in all regions in 2016, except for the South, and the highest rates recorded were in the North and Northeast Region of the country ^(22)^.

There is a concern about the repercussion of the pandemic caused by SARS-CoV infection 2 in childhood. Although the pediatric population is less affected and often asymptomatic than the adult population, data on risk factors related to mortality in children still have limitations^(7-8)^.

In Brazil, according to the data presented in the results of this study, the mortality rate due to COVID-19 in the infant age group is higher among children aged 1 to 4 years, followed by children under one year of age. These findings may be related to the severity with which the disease has presented itself in this population. In studies conducted in China^(23)^ and in Italy^(24)^, the severity of infection and complications was higher in younger children, especially in infants (under one year) and preschoolers (from 1 to 5 years). It allows us to infer that belonging to those age groups can be a risk factor for the severity of the disease and consequent mortality among children.

A Chinese study that analyzed 2,135 cases of the disease through clinical characteristics, laboratory tests, and X-rays of pediatric patients showed that children under one year old were more vulnerable to infection. The proportions of severe and critical cases were 10.6% in children under one year, 7.3% in children aged 1 to 5 years, 4.2% in the age group 6 to 10 years, 4.1% in those aged 11 to 15 years, and 3% in those over 16 years^(23)^.

A study conducted in Italy found equivalent results, in which children under one year of age with the presence of underlying disease constituted a risk factor for the severity of the disease. The study considered pneumonia, dyspnea due to hypoxia, tachypnea, and requiring hospitalization cases as severe; and individuals who developed severe pneumonia, acute respiratory distress syndrome, septic shock, and/or multiple organ dysfunction and who remained hospitalized in intensive care were considered critical cases^(24)^. The hospitalization rate was higher among children under one year old (36.6%) and between 2 and 6 years old (12.8%). The rate of hospitalization in the intensive care unit (ICU) was higher among children aged 2 to 6 years (9.5%), these age groups, which corroborate those presented in this study^(24)^.

Regarding the need for ICU admission, an American study^(25)^ pointed out that children under the age of one year represented the highest percentage of hospitalization among pediatric patients with COVID-19. Among ninety-five diagnosed children under the age of one, 59 (62%) were hospitalized, and five of them required intensive care. It converges with the findings of another American study^(26)^, in which one in three hospitalized children was admitted to the intensive care unit.

Internationally, children under one year old with a history of prematurity or other chronic health conditions present more severe forms of the disease and require more hospitalizations and admission in intensive care units when compared to older and younger children. In the Brazilian scenario, people face difficulty in accessing health services and ICU beds^(27)^. Thus, the study suggests an association between severity in this age group, the need for hospitalization and intensive care, and the higher mortality rate among children aged 1 to 4 years and younger than one year old.

Therefore, COVID-19 culminates in our country at the height of an economic crisis that has plagued it since 2015, and it is in this context that sanitary and economic disparities stand out. The pandemic in Brazil manifests these inequalities in coping with both health and economic tribulations^(28)^.

In this scenario, ethnic-racial issues have been a subject in discussions on social determinants of health in Brazil. Race and social and economic conditions influence the health of Brazilians^(29)^. Despite infant mortality, studies have shown higher rates in black and brown children when compared to white and yellow children^(29-30)^. However, there is a lack of studies on the differences between Indigenous and non-Indigenous children^(31)^. A current ecological study conducted in Rio de Janeiro showed that the cumulative incidence rates of COVID-19 are influenced by income, suggesting that access to exams is occurring unevenly, that is, testing for COVID-19 is being more widespread in more affluent regions of the city^(32)^.

The present study demonstrated a marked disparity in the mortality rate from COVID 19 in Indigenous children in all age groups described. The findings mentioned here confirm other studies that showed high death rates of Indigenous children due to various causes^(31,33)^. In this pandemic context, the considerable number of cases can be attributed to the low socioeconomic level and the living conditions of the Indigenous population, which, for the most part, resides in agglomerations, has large families, insufficient basic sanitation, and current proximity to urban life. Still, the lack of state protection, through the depreciation of scientific evidence, can be an aggravating factor for these population groups^(34)^, as well as the disregard for the underreporting of COVID-19 cases^(35)^.

In addition to the age and race issues, this study highlights the geographic disparity in the number of death cases among children due to COVID-19. The mortality rate is higher in the Northern (especially Roraima and Pará) and northeast states (especially the States of Pernambuco and Sergipe) in all age groups analyzed.

The social and territorial inequality of our country is evident in the number of child mortality by COVID-19. The State of Pernambuco, even before the pandemic, already showed geographic disparities concerning infant mortality. A study conducted through spatial analysis revealed a correlation between the poverty rate with the infant mortality rate, especially in the municipalities that make up the coast^(36)^.

The North and Northeast regions suffer from inequities in access to health. The increase in the number of ICU beds during the pandemic remarks the regional inequalities in their distribution and allocation in the country, among them neonatal and pediatric intensive care. The increased number of beds in the country was from 46,045 (total number pre-pandemic) to 60,265 (total number post-pandemic). However, of this new 14,220 beds, only 3,104 are of free and accessible access for the entire population through the SUS^(37)^.

When analyzing the availability of beds by region, inequalities become even more explicit. The Southeast Region concentrates 51% of the National beds while the North (5.2%) and Midwest (8.5%) do not reach even 10% of the total Brazilian beds^(37)^.

The Northern Region has a population contingent of 18.43 million people who dispute 1,793 SUS beds of ICU. About 90% of the population depends exclusively on the public health system: in absolute numbers, it means that there is approximately one SUS bed for every 9,325 people. The population of the Northeast Region is 57.07 million people, of which 88.43% depending on the SUS and dispute 5,968 beds, which equals one bed for every 8,456 people^(37)^. Social and territorial inequalities are even more evident in the pandemic; however, they were already accentuated before it, which points to a reflection on the likely future impacts on the health and life of Brazilian children.

An ecological study conducted in nine states of the Northeast Region identified that the lethality due to COVID-19 was 8% in children and adolescents from 0 to 19 years, with a prevalence of 321/100 thousand inhabitants^(38)^. This study highlighted the States of Piauí, and Rio Grande do Norte with high prevalence and lethality, respectively.

Although children are not the most clinically affected by the virus, they are being hit and may suffer consequences, as well as impacts on IMT in the country, because of the social and economic losses involved in the pandemic. A model study^(38)^ with hypothetical scenarios and already reduced maternal and child health services, which estimated maternal and children under five years of age deaths due to the impact of COVID-19, noticed that the influence on the reduction of health coverage (9.8% to 18.5%) in six months would result in an additional 253,500 additional infant deaths. In an even more severe scenario, reducing coverage from 39.3% to 51.9% over six months would result in additional 1,157,000 child deaths. This additional would represent a 9.8% to 44.7% increase in the number of deaths of under-5s per month in the 118 countries. Crises, workforce reductions, lack of supplies, difficulties in access and coverage can have repercussions on the conditions of care for family planning, prenatal care, childbirth care, postnatal care, early childhood vaccinations, curative care, and prevention measures for children^(39)^.

### Study limitations

The study limitations include the use of secondary data for its development, which are affected by problems related to underreporting, and notification errors, such as in the recording of the variables age and color/race. It is a usual limitation in studies of child mortality, according to evaluations of underreporting already performed in works with method and theme^(35)^.

### Contributions to the fields of Health or Public Policy

This study will contribute to the importance of appropriate actions to prevent COVID-19 mortality among children and adolescents. The fact that it found higher rates for the age groups under five years old should serve as attention, including for the policies of reopening daycare centers and pre-schools, being necessary specific protocol actions for this age group. Higher mortality rates among race-colored groups should also be used to think about creating policies focused primarily on the most vulnerable populations and the most frequent regions.

Regarding care, the data presented show the importance of preventive action. Particularly, health professionals working in primary health care, such as childcare programs and vaccination rooms, need to be attentive and guide family caregivers regarding the risks of infection in children under one year old. Likewise, professionals working with Indigenous populations need to be aware of the results of this study and consider them in planning their daily practice.

## CONCLUSION

The study concluded that the mortality rate among children and adolescents due to COVID-19 is a crucial indicator to be considered in epidemiological studies and control of the disease, as well as in its prevention and control policies. The average rate was 1.34 deaths per one hundred thousand for the group studied, highlighting a higher frequency and mortality rate in children aged between 1 and 4 years of age. Children under five years old represent just over half of the total deaths due to COVID-19 in the studied population and show the highest mortality rates.

It also evidenced the existence of social and territorial inequalities in the mortality of children and adolescents due to complications derived from COVID-19 in Brazil. The most vulnerable populations to death were brown and Indigenous people, especially residents in the North and Northeast Regions of Brazil.

The development of other qualitative approach studies with this population, as well as the health follow-up of the survivors to analyze the possible sequelae arising from the infection, is strongly recommended.
